# Reversal of increased mammary tumorigenesis by valproic acid and hydralazine in offspring of dams fed high fat diet during pregnancy

**DOI:** 10.1038/s41598-019-56854-5

**Published:** 2019-12-30

**Authors:** F. de Oliveira Andrade, N. M. Nguyen, A. Warri, L. Hilakivi-Clarke

**Affiliations:** 1 Department of Oncology, Georgetown University, Washington, DC USA; 2 Institute of Biomedicine, University of Turku Medical Faculty, FI-20014 Turku, Finland

**Keywords:** Breast cancer, Breast cancer

## Abstract

Maternal or paternal high fat (HF) diet can modify the epigenome in germ cells and fetal somatic cells leading to an increased susceptibility among female offspring of multiple generations to develop breast cancer. We determined if combined treatment with broad spectrum DNA methyltransferase (DNMT) inhibitor hydralazine and histone deacetylase (HDAC) inhibitor valproic acid (VPA) will reverse this increased risk. C57BL/6 mouse dams were fed either a corn oil-based HF or control diet during pregnancy. Starting at age 7 weeks, female offspring were administered 3 doses of 7,12-dimethylbenz[a]anthracene (DMBA) to initiate mammary cancer. After last dose, offspring started receiving VPA/hydralazine administered via drinking water: no adverse health effects were detected. VPA/hydralazine reduced mammary tumor multiplicity and lengthened tumor latency in HF offspring when compared with non-treated HF offspring. The drug combination inhibited DNMT3a protein levels and increased expression of the tumor suppressor gene *Cdkn2a/p16* in mammary tumors of HF offspring. In control mice not exposed to HF diet *in utero*, VPA/hydralazine increased mammary tumor incidence and burden, and elevated expression of the unfolded protein response and autophagy genes, including HIF-1α, NFkB, PERK, and SQSTM1/p62. Expression of these genes was already upregulated in HF offspring prior to VPA/hydralazine treatment. These findings suggest that breast cancer prevention strategies with HDAC/DNMT inhibitors need to be individually tailored.

## Introduction

The causes of breast cancer remain largely unknown. While exposure to DNA damaging agents such as radiation and carcinogens are likely required for initiation^1^, this exposure alone may not be sufficient to drive clinically detectable breast cancer. For example, women exposed at an early age to radiation from the atomic bomb^2^, or to ionizing therapeutic^3^ or diagnostic radiation^4^ experience a 2–9-fold increase in breast cancer risk, but <20% of these women develop breast cancer^3^. Family history of breast cancer, especially having inherited germline BRCA1/2 mutation, greatly increases lifetime breast cancer risk. However, only 10% of all breast cancer patients have a strong family history of breast cancer^5^. Other factors that pre-program an increased susceptibility to breast cancer include *in utero* exposures to estrogenic compounds, such as maternal intake of the synthetic estrogen diethylstilbestrol (DES)^6,7^ and maternal exposure to the endocrine disrupting compound dichlorodiphenyltrichloroethane (DDT) during pregnancy^8^. Excess maternal weight gain during pregnancy and high birthweight in both rats^9^ and humans^10,11^ also increase susceptibility to breast cancer. We^12–14^ and others^15–18^ have found an increase in mammary cancer risk following an *in utero* exposure to a high fat (HF) diet in preclinical animal models. These maternal exposures may also affect key reproductive factors, such as induce an early puberty onset^12,19^, which are linked to an increased breast cancer risk^20^.

Many of the maternal exposures linked to an increased breast cancer susceptibility among daughters induce epigenetic modifications in the fetal cells without affecting DNA sequence. These epigenetic modifications are heritable, persist into adulthood^21,22^, and could lead to either the silencing of key tumor suppressor genes or activation of oncogenes^23^. We found an increase in DNA methyltransferase (DNMT) expression in mammary glands of F1-F3 generation offspring of dams exposed to ethinyl estradiol (EE2) during pregnancy^13^. However, the causality of epigenetic modifications resulting from *in utero* exposures and increased breast cancer risk has not been studied.

Since the epigenetic changes induced by histone deacetylases (HDACs) and DNMTs are potentially reversible^24^, we posited that treating adult HF offspring with broad spectrum inhibitors of DNMTs and HDACs might prevent their increased mammary cancer risk, perhaps by reversing the downregulation of tumor suppressor genes. Interactive and complex functional cross-talk between HDAC and DNMT activities makes a combination of both HDAC and DNMT inhibitors more effective in inhibiting the growth of different cancers in experimental models than either inhibitor alone^25–27^. Further, since our goal is to prevent healthy women at high risk of breast cancer from developing this disease, the HDAC and DNMT inhibitors to be used need to be safe and not toxic. Thus, the DNMT inhibitors azacytidine (Vidaza; Celgene) and decitabine (5-aza-2-deoxycytidine, 5-Aza-CdR) (Dacogen; SuperGen) that are used to treat myeloid blood cancers^28^ are toxic^29^ and not suitable as preventive drugs. We chose to use valproic acid that inhibits class I HDACs^30,31^, and hydralazine that suppresses DNMT1 and DNMT3a activities^32^. VPA was developed for the treatment of neurological diseases, such as epilepsy and migraine, and it is effective in the treatment of bipolar disease. Hydralazine is an antihypertensive drug. Preclinical studies and clinical trials have combined these two drugs with standard therapies to reverse epigenetic changes^33^ and treat leukemia^34^ and some advanced solid cancers^35–37^. These drugs can also prevent the development of drug resistance or resensitize therapy-resistant cancer cells *in vitro* and in preclinical models^33,38^. Since VPA and hydralazine can be given chronically, long-term use of the combination is realistic for a cancer prevention strategy. However, VPA is teratogenic^39^ and cannot be used by pregnant mothers. Therefore, we tested the possibility that an adult exposure to VPA/hydralazine prevents the increased risk of mammary cancer associated with an *in utero* exposure to a HF diet.

Our results indicated that VPA/hydralazine reduced mammary tumor multiplicity and lengthened tumor latency in the HF offspring. However, in the control offspring treatment with VPA/hydralazine increased both mammary tumor incidence and tumor burden. The opposing effects in *in utero* HF or control diet exposed rats were linked to different effects on the expression of tumor suppressor and oncogenes. Our findings suggest that similar to the idea that patients with different gene expression signatures in their breast cancers need an individualized treatment plan^40^, approaches to prevent breast cancer should be tailored to reflect a woman’s breast cancer susceptibility factors.

## Results

### Effect of VPA/hydralazine on body weight

In this study, pregnant C57BL/6NTac mice were fed either a high fat diet containing high levels of n-6 polyunsaturated acids (PUFAs) in corn oil, or a control diet. The control diet also had corn oil as the main fat source, but the percentage of fat was lower in the control (17%) than HF diet (43%). When offspring were adults, some of them received a treatment with VPA and hydralazine via drinking water, starting one week after mice received the last of three doses of 7,12-dimethylbenz[a]anthracene (DMBA) to initiate mammary tumorigenesis. The most notable side-effect of VPA in individuals taking this drug is weight gain^41,42^. No excessive weight gain was seen in the control offspring when VPA was given in combination with hydralazine. Among the HF offspring, treatment with VPA/hydralazine reduced body weight gain when compared with non-treated HF offspring (p = 0.037; 2-way ANOVA, p = 0.018) (Supplementary Fig. 1). Since HF and control diets fed to pregnant dams are isocaloric, no differences in body weights occurred between the non-treated control and HF offspring at any stage of their life-cycle^14^.

### Effect of VPA/hydralazine on mammary tumorigenesis

#### Tumor incidence

**Figure 1:**
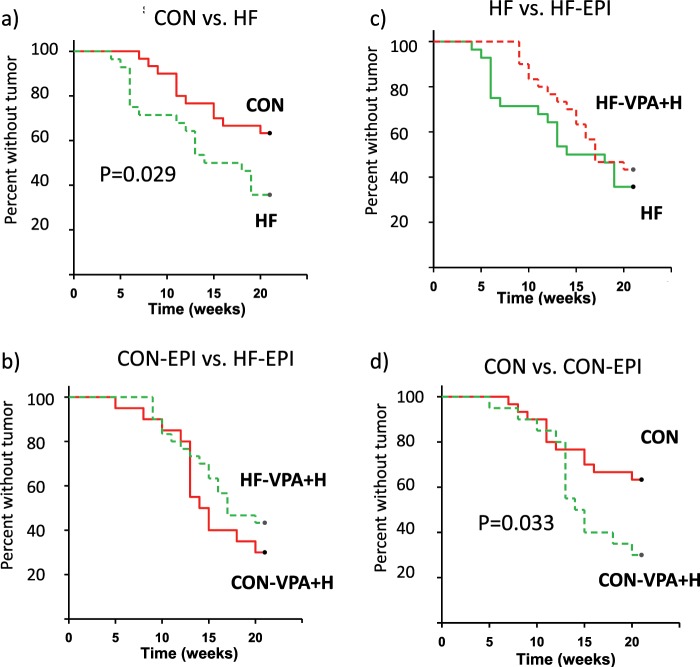
Effect of valproic acid (VPA) and hydralazine on mammary tumor incidence. (**a**) A significantly higher number of offspring of high fat (HF) fed dams (n = 28) developed mammary tumors during the 20 week tumor monitoring period than control (CON) offspring (n = 30). (**b**) The difference was eliminated by treatment with VPA/hydralazine (abbreviated as EPI) (n = 18 in CON-EPI and n = 30 in HF-EPI). (**c**) VPA/hydralazine treatment non-significantly delayed mammary tumor development in HF offspring. (**d**) VPA/hydralazine treated CON mice developed more mammary tumors than non-treated CON mice. For statistical analysis, Kaplan-Maier Survival Analysis was performed; p-values given in the figures.

Of control mice exposed to medroxyprogesterone acetate (MPA), followed by three doses of DMBA, 36.7% developed malignant tumors, 20.0% developed only benign tumors, and 43.3% did not develop any tumors during a 20-week tumor monitoring period. The incidence of malignant tumors was 64.3%, benign tumors 14.3%, and no tumors in 21.4% of the HF offspring. The percentage of malignant tumors was significantly higher (χ^2^ test, p < 0.001), whereas the percentage of mice not developing any tumors was significantly lower (p = 0.001) in the HF group compared with the controls. Kaplan-Meier survival analysis confirmed that a significantly higher number of HF offspring developed malignant mammary tumors than control offspring during the tumor monitoring period (p = 0.029) (Fig. 1a).

Treatment in adulthood with VPA/hydralazine eliminated the difference in mammary tumor incidence between untreated control and HF offspring (Fig. 1b). This difference did not reflect an effect of VPA/hydralazine in the HF offspring because the incidence of malignant tumors in these animals was only reduced by 12% (from 64.3% to 56.7%) with VPA/valproic acid (Fig. 1c). A two-fold increase in mammary tumor incidence was seen in the control offspring treated with VPA/hydralazine (75.0%), when compared with their non-treated controls (36.7%) (p < 0.001). Kaplan-Meier survival analysis also indicated a significant increase in the number of animals that developed mammary tumors when receiving VPA/hydralazine in the control group (p = 0.033) (Fig. 1d).

When treated with VPA/hydralazine, only 5% of control mice and none of the HF offspring developed benign tumors. Thus, the EPI treatment increased the proportion of malignant tumors (χ^2^ test, control group: p = 0.003; HF: p < 0.001). However, as shown in Fig. 1d, an increase in the total number of malignant tumors was seen only in the control group.

#### Tumor latency and multiplicity

**Figure 2:**
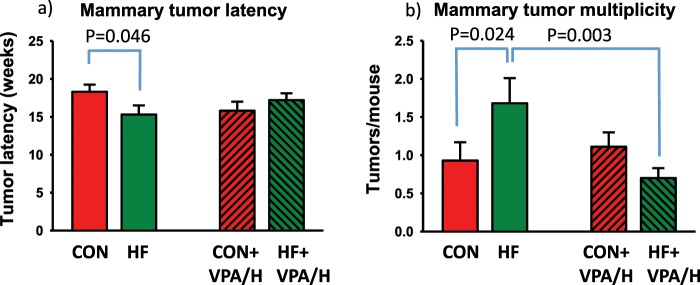
Mammary tumor latency and multiplicity during valproic acid (VPA) and hydralazine treatment. (**a**) Mammary tumor latency was significantly (p-value obtained by Holm-Sidak post-hoc analysis shown in figure) shorter in high fat (HF) offspring, compared with control (CON) offspring. Treatment with VPA/hydralazine non-significantly shortened tumor latency in CON offspring, but had opposite effect on HF offspring (p for interaction = 0.047). (**b**) Mammary tumor multiplicity (number of tumors per mice) was significantly higher in HF offspring, compared with CON offspring. Treatment with VPA/hydralazine significantly reduced tumor multiplicity in HF offspring (p for interaction = 0.027). Means and SEM of 18–30 mice per group are shown.

HF offspring developed mammary tumors significantly earlier than control offspring (p = 0.046). Treatment with VPA/hydralazine delayed mammary tumor development in the HF offspring, whilst in the control group VPA/hydralazine accelerated tumor development (p for interaction = 0.047) (Fig. 2a). Tumor multiplicity also was affected by maternal diet: multiplicity was significantly higher in HF offspring than in controls (p = 0.024) (Fig. 2b). Treatment with VPA/hydralazine significantly reduced tumor multiplicity in HF offspring (p = 0.003) but had no effect on control offspring (p for interaction = 0.020).

#### Tumor burden

**Figure 3:**
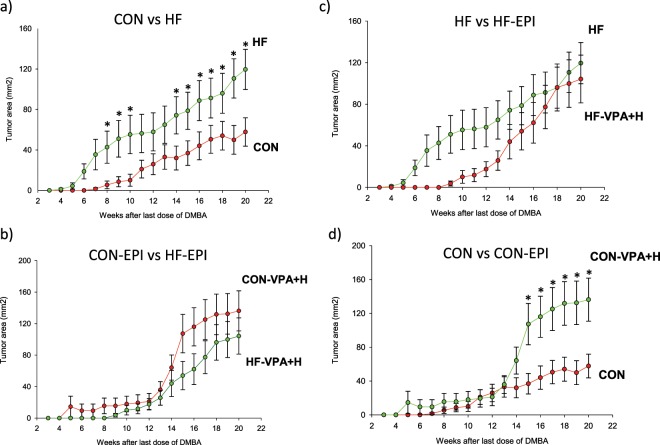
Effect of valproic acid (VPA) and hydralazine on mammary tumor burden. (**a**) Mammary tumor burden, assessed by measuring tumor volume (diameter × width), was significantly higher (p = 0.029) in the offspring of high fat (HF) fed dams (n = 28) during 20 week tumor monitoring period than in control (CON) offspring (n = 30). (**b**) The difference was lost (p = 0.16) by treatment with VPA/hydralazine (abbreviated as EPI) (n = 18 in CON-EPI and n = 30 in HF-EPI). (**c**) VPA/hydralazine treatment did not change (p = 0.13) mammary tumor burden in HF offspring. (**d**) VPA/hydralazine treated CON mice had significantly higher (p = 0.027) mammary tumor burden than non-treated CON mice. Means and SEM of tumor volume at each tumor monitoring week are shown; * indicates statistical significance during weeks when it reached p < 0.05. Differences were analyzed according to 2-way Repeated Measures ANOVA followed by Holm Sidak test.

Tumor burden represents the number of tumors per mice and their combined size. HF offspring exhibited a significantly higher tumor burden than control offspring (repeated measures ANOVA; p = 0.029) (Fig. 3a). This difference in tumor burden was not seen in HF and control offspring treated with VPA/hydralazine (Fig. 3b**)**. VPA/hydralazine treatment did not change tumor burden in the HF, compared with non-treated HF offspring (Fig. 3c). In the control offspring, VPA/hydralazine treatment significantly increased mammary tumor burden (p = 0.027) (Fig. 3d).

In summary, VPA/hydralazine treatment reversed the increased tumor multiplicity in HF offspring, and non-significantly delayed their tumor development, but did not reduce mammary tumor incidence. In marked contrast, VPA/hydralazine increased both mammary tumor incidence and burden in the control offspring.

### Effect of VPA/hydralazine on tumor suppressor gene expression in mammary tumors

**Figure 4:**
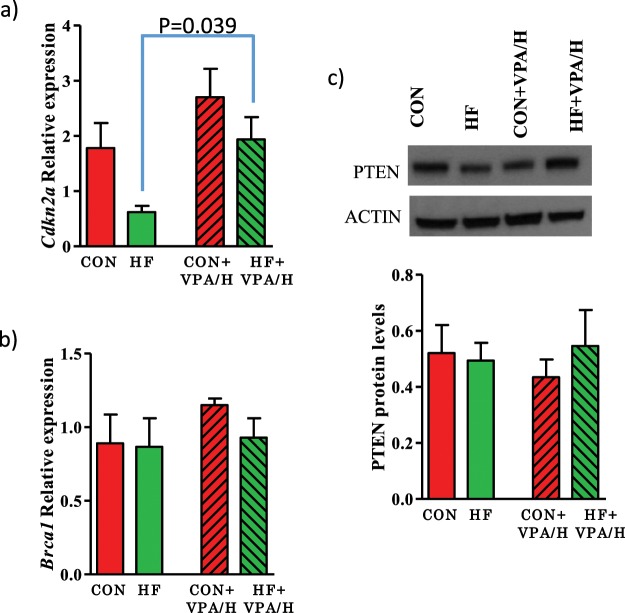
Effect of valproic acid (VPA) and hydralazine on tumor suppressor gene expression in mammary tumors. (**a**) *Cdkn2a* expression was significantly lower in HF offspring than in controls (p for group = 0.034), and significantly increased by VPA/hydralazine (p for treatment = 0.015). Post-hoc analysis showed that the effect of VPA/hydralazine was significant in HF offspring (p-value shown in figure). Neither (**b**) *Brca1* nor (**c**) PTEN expression was altered in mammary tumors. Means and SEM of 4–7 malignant adenocarcinomas per group are shown.

We determined whether some of the commonly silenced breast cancer tumor suppressor genes were differentially expressed in the mammary tumors between control and HF offspring, specifically BRCA1, CDKN2A, and PTEN. Expression of *Cdkn2a* was lower in the HF offspring; treatment with VPA/hydralazine significantly increased this expression (p = 0.04, p for VPA/hydralazine treatment = 0.015) (Fig. 4a). The other tumor suppressor genes studied were not differentially expressed or affected by VPA/hydralazine treatment in the HF offspring (Fig. 4b,c, Supplementary Fig. 2). We also determined if any of the tumor suppressor genes found to be down-regulated in the mammary glands of F1 and F3 generation offspring of HF fed dams in our previous study^14^ – *Igfbp6*, *Oas3a*, *p21*, *Slfn1 or Zbp1* – were affected by VPA/hydralazine treatment in mammary tumors: they were not (Supplementary Fig. 3).

### Effect of VPA/hydralazine on estrogen receptor levels in mammary tumors

Methylation of the estrogen receptor (ER) is more common in young girls with a strong family history of breast cancer, but who are not carriers of BRCA germline mutations, than in girls without such family history^43^. Further, low ER expression in human breast cancer cells can be reversed by treatment with DNMT and HDAC inhibitors^44^. We assessed if ERα or ERβ were differentially expressed in mammary tumors of control and HF offspring. No statistically significant differences between the non-treated control and HF offspring were observed. There was a non-significant tendency for VPA/H to upregulate ERα and ERβ in both the control and HF offspring (Supplementary Figs. 4 and 5).

### Effect of VPA/hydralazine on CpG island methylation of CDKN2A in mammary tumors

**Figure 5:**
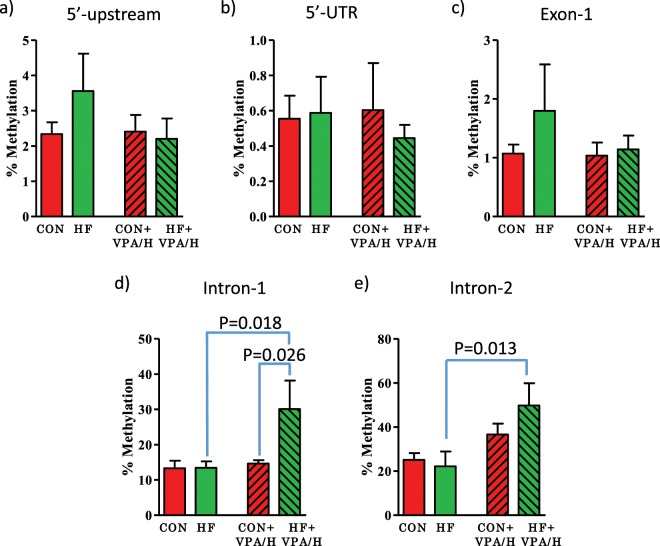
Effect of valproic acid (VPA) and hydralazine on CpG island methylation of *Cdkn2a* in mammary tumors. Percentile of CpG island methylation in (**a**) 5′-upstream promoter region, (**b**) 5′-UTR, (**c**) Exon-1, (**d**) Intron-1, and (**e**) Intron-2 of *Cdkn2a* gene in control and high fat (HF) offspring, with or without VPA/hydralazine treatment. Treatment with VPA/hydralazine increased methylation in HF offspring in intron-1 (p for treatment = 0.058) and intron-2 (p for treatment = 0.013). Means and SEM of 4 malignant adenocarcinomas per group are shown. P-values in figure indicate differences by Holm-Sidak post-hoc test.

Since Cdkn2a emerged as the downregulated tumor suppressor potentially linked to increased breast cancer risk in the HF offspring, we measured changes in CpG island methylation across the *Cdkn2a* gene, including within the gene body and promoter regions upstream from the gene transcription start site (Supplementary Fig. 6). While not statistically significant, VPA/hydralazine decreased the elevated DNA methylation of *Cdkn2a* in both the promoter region and 1^st^ exon in HF group (Fig. 5). However, after treatment with VPA/hydralazine, a significant increase in DNA methylation was observed in the gene body in the HF group in introns 1 (p = 0.018, p for VPA/hydralazine = 0.058) and 2 (p = 0.013, p for VPA/hydralazine = 0.013), compared with non-treated HF offspring (Fig. 5**)**. These findings are consistent with earlier studies showing that methylation of gene body increases mRNA expression^45,46^. Nevertheless, the increase in CpG methylation in gene body with VPA/hydralazine treatment was unexpected.

### Effect of VPA/hydralazine on DNMTs and HDACs in mammary tumors

**Figure 6:**
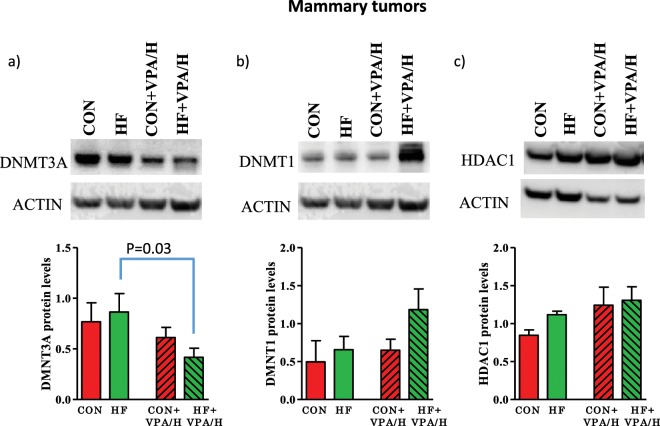
Effect of valproic acid (VPA) and hydralazine on DNMT and HDAC protein levels in mammary tumors. Treatment with VPA/hydralazine decreased the expression of (**a**) DNMT3A (p = 0.046 for treatment). No changes in the levels of (**b**) DNMT1 or (**c**) HDAC1 were seen. P-values obtained in Holm-Sidak post-hoc test are shown in figure. Means and SEM of 4–7 tumors per group are shown.

We then determined if the increase in gene body methylation by VPA/hydralazine in the HF offspring reflected changes in DNMT levels. VPA/hydralazine significantly downregulated protein expression of DNMT3a in the mammary tumors of HF offspring (p = 0.03 Holm-Sidak test after 2-way ANOVA, p for treatment = 0.046) (Fig. 6a, Supplementary Fig. 7). This observation is consistent with an earlier study showing that hydralazine inhibits DNMT3a^32^. Prior to VPA/hydralazine treatment, DNMT3a, DNMT1 (Fig. 6b) or HDAC1 (Fig. 6c) expression was not different in the mammary tumors of HF offspring, compared with controls.

### Effect of VPA/hydralazine on markers of endoplasmic reticulum stress and autophagy in mammary tumors

**Figure 7:**
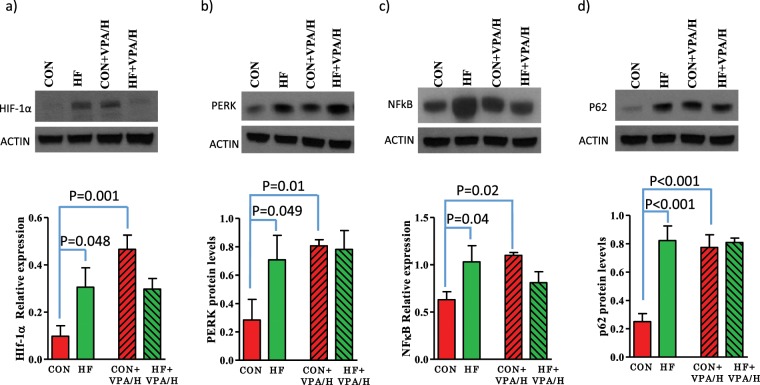
Effect of valproic acid (VPA) and hydralazine on markers of endoplasmic reticulum stress and autophagy in mammary tumors. Compared with control offspring, *in utero* HF exposed offspring exhibited increased protein levels of (**a**) HIF-1α, (**b**) PERK, (**c**) NFκB, d) SQSTM1/P62. Treatment with VPA/hydralazine increased the levels in control group, but not in HF offspring, of (**a**) HIF-1α (p = 0.001 for interaction), (**b**) PERK (p = 0.01 for treatment), (**c**) NFκB (p = 0.011 for interaction), (**d**) SQSTM1/P62 (p = 0.005 for treatment, and p = 0.003 for interaction). P-values obtained in Holm-Sidak post-hoc test are shown in figure. Means and SEM of 4–7 tumors per group are shown.

We next determined if expression of oncogenes previously linked to breast cancer is altered in VPA/hydralazine treated mice. We first measured the endoplasmic reticulum (EnR) stress/unfolded protein response (UPR) and genes known to affect the UPR. UPR is activated in cells undergoing malignant transformation^47^, for example as a consequence of hypoxia. HIF1α, a master transcriptional regulator of the response to hypoxia, was upregulated in the mammary tumors of HF offspring (p = 0.048). Treatment with VPA/hydralazine increased protein levels in the control (p = 0.001), but not in HF offspring (p for interaction = 0.008) (Fig. 7a, Supplementary Fig. 8). Of the three UPR signaling arms, PERK was upregulated in HF offspring (p = 0.049), as was the PERK downstream target NFκB (p = 0.04). NFκB is a key regulator of inflammatory responses. Treatment with VPA/hydralazine increased the levels of PERK (p = 0.01) and NFκB (p = 0.02) in control offspring to the levels seen in HF offspring, but did not further affect the expression of these genes in the HF offspring (Fig. 7b,c, Supplementary Fig. 8**)**.

Expression of p62, which acts as an autophagy cargo receptor by linking ubiquitin-tagged protein aggregates to autophagosomes for degradation, also was elevated in the mammary tumors of HF offspring. As with HIF1α, PERK, and NFκB, treatment with VPA/hydralazine increased p62 protein levels in the control group (p < 0.001) (Fig. 7d, Supplementary Fig. 8), and the elevated levels of p62 in the HF offspring were not further altered by VPA/hydralazine. In addition to being linked to autophagy, p62 also enhances breast cancer stem-like properties^48^, and increases breast cancer cell proliferation^49^. Further, elevated p62 expression is associated with a shorter disease-free survival in breast cancer patients^50^. Thus, the increased p62 levels in the tumors of VPA/hydralazine treated control offspring and non-treated HF offspring likely contribute to increased mammary tumorigenesis.

## Discussion

In preclinical models, an increase in mammary cancer risk following an *in utero* exposure to a HF diet or to endocrine disrupting chemicals is associated with epigenetic changes. These changes include alterations in DNA methylation and histone modifications in the mammary gland^13,21,22^. We investigated here if the increased mammary cancer risk and epigenetic changes in *in utero* HF exposed animals are reversible by treating mice with the broad spectrum HDAC and DNMT inhibitors VPA and hydralazine, respectively. Earlier studies indicate that a combination of HDAC and DNMT inhibitors, alone or together with other therapies, reverse epigenetic alterations and inhibit growth of cancer cells *in vitro*^25,26^ and in patients with advanced cancer^35–37^. In our earlier study, HDAC inhibiting VPA and DNMT inhibiting hydralazine prevented tamoxifen resistance and local mammary cancer recurrence in animals exposed to the endocrine disrupting chemical EE2 *in utero*^38^. The present study represents the first time the outcomes of HDAC and DNMT inhibitor treatments were combined with the knowledge of prior epigenetic changes in mammary tumors. The results showed that VPA/hydralazine treatment significantly reduced tumor multiplicity and lengthened tumor latency in the HF offspring.

However, VPA/hydralazine treatment adversely affected the control offspring. Controls receiving these drugs exhibited an increase in mammary tumorigenesis over their untreated counterparts. Thus, the benefits of this therapy were limited to mice exposed to an *in utero* HF environment. Similar data were obtained in our earlier study in which VPA/hydralazine increased mammary cancer recurrence in control offspring, whilst reduced recurrence in offspring of dams treated with the synthetic estrogen EE2 during pregnancy^38^. These results underline the importance of patient stratification based on their potential epigenetic changes, and the urgent need for epigenetic biomarkers for assessing those changes.

To understand why VPA/hydralazine treatment has opposite effects on HF and control offspring, we investigated their effects on the expression of key tumor suppressor genes and oncogenes associated with breast cancer risk. In breast cancer patients, tumor suppressor genes that are frequently downregulated by methylation include BRCA1^51^, CDKN2A^52^, and PTEN^53^. Of these, *Cdkn2a* mRNA expression was downregulated in the mammary tumors of HF offspring, and treatment with VPA/hydralazine reversed this downregulation. Consistent with this result, earlier studies have reported that hydralazine treatment reactivates *CDKN2A* in human breast cancer cells^54^ and in human breast tumors^55^. Since epigenetic repression of *CDKN2A* has been identified as a key driver of malignant transformation in the breast^52^, maternal HF intake during pregnancy may increase an offspring’s breast cancer risk by epigenetically silencing this tumor suppressor gene.

It is not clear how treatment with VPA/hydralazine reversed the downregulation of *Cdkn2a*. No significant differences in DNA methylation in the promoter region, 5′-UTR or the 1^st^ exon of *Cdkn2a* were seen in the mammary tumors between the control and HF offspring. The decreased methylation of these two locations following VPA/hydralazine treatment did not reach statistical significance. Unexpectedly, VPA/hydralazine treatment significantly increased methylation of *Cdkn2a* in introns 1 and 2. Since DNA methylation in the gene body can increase gene expression^45,56^, the observed increase in intron methylation in HF offspring is consistent with increased mRNA expression of *Cdkn2a* by VPA/hydralazine.

We cannot exclude the possibility that the results obtained in control offspring reflected other properties of VPA/hydralazine, such as their effects on neurological end-points or blood pressure. However, these effects have not been linked to increased breast cancer risk. A more plausible explanation is that VPA/hydralazine upregulated several oncogenes linked to increased breast cancer risk in the control mice that were already upregulated in non-treated HF offspring. We found several cancer-promoting UPR-linked genes to be upregulated in the mammary tumors of control offspring treated with VPA/hydralazine, including HIF-1α^57^, NFκB^58^, PERK^59^, and p62^60^. These findings are consistent with earlier studies showing that hydralazine upregulates HIF-1α in smooth muscle cells^61^. Since sustained, non-lethal levels of UPR permit cancer cells to survive and continue to grow despite hostile internal and external conditions that include nutrient and lipid deprivation and hypoxia^62^, HDAC/DNMT inhibitors may increase breast cancer risk in individuals not exposed to *in utero* manipulations by upregulating UPR.

Given how time consuming and expensive it is to develop new drugs, repurposing FDA-approved drugs with known low toxicity has gained increased attention and many are being tested for effectiveness in cancers. VPA/hydralazine are among these drugs^35–37^. We now show that the earlier onset of mammary cancer and the development of multiple mammary tumors in female offspring exposed *in utero* to a maternal HF diet can be reversed by treating adult offspring with VPA/hydralazine. However, prior to translating these findings into the clinic it is critical to identify women who will benefit from taking VPA/hydralazine, and those who may be harmed. VPA/hydralazine promoted mammary tumorigenesis in the control offspring in this and another study from our group^38^. Population-based case-control studies have failed to show that VPA reduces cancer risk. In fact, the incidence of lung^63^ and colorectal cancers^64^ is significantly higher among individuals using VPA than non-users. Women who could potentially benefit from VPA/hydralazine treatment are daughters of mothers who consumed a HF diet during pregnancy. Our unpublished data would extend that group to include women exposed to endocrine disrupting compounds (EDCs) *in utero*. Importantly, it should be investigated if those women not exposed to high fat diet or EDCs *in utero*, and who are taking VPA to treat epilepsy, bipolar disorder, or migraine, and/or taking hydralazine to treat hypertension, experience an increased risk of developing breast cancer.

Several foods and bioactive nutrients modify the epigenome^65,66^. However, epidemiological studies have mostly generated conflicting findings regarding the efficacy of any of these foods or nutrients in breast cancer prevention. One reason why attempts have failed to link them to reduced breast cancer risk might be that we have assumed nutrients with epigenetic properties are equally effective in all women. Based on the findings of our study, administration of any compound that modifies the epigenome, including VPA/hydralazine, may need to be individually tailored to be used for the prevention of breast cancer.

## Materials and Methods

All methods described in this manuscript were performed in accordance with the relevant guidelines and regulations, as described by the guidelines of Scientific Reports at www.nature.com/srep/policies/index.html#experimental-subjects.

### Breeding and dietary exposure

Four-week-old male and female C57BL/6NTac mice were obtained from Taconic Biosciences (Germantown, NY) and housed in standard rodent housing at constant temperature, humidity, and a 12-hr light/dark cycle at Georgetown University’s Department of Comparative Medicine (DCM), in accordance with all institutional and federal regulations.

When mice were 7 weeks of age, they were mated by housing 2 females together with 1 male per cage. Pregnancy was verified by the presence of a mucus copulatory plug in the vaginal opening; this day was assigned as gestation day (GD) 0. On GD10, the pregnant dams were divided into two groups and fed either a HF diet containing 43% energy from fat (per 100 g of food contained 18% corn oil, CO, and 1% soybean oil, SBO), or continued on the control diet (17% energy from fat, 6% CO per 100 g diet, 1% SBO)^14^. The HF diet was made isocaloric with the control diet by replacing some carbohydrates with non-energy containing cellulose (fiber). Both diets contained the same amount of protein, and vitamins and minerals. Upon giving birth on GD20-21, dams and their offspring in both groups were fed only control diet.

### Mammary tumorigenesis

To induce mammary tumors, female C57BL6/NTac mice were first primed with 15 mg/kg of medroxyprogesterone acetate (MPA) (Greenstone, Peapack, NJ) at PND42. One week later, 1 mg of 7,12-dimethylbenz[a]anthracene (DMBA) (Sigma, St. Louis, MO), in 1 ml of peanut oil, was administered by oral gavage. DMBA administration was repeated 2 additional times at one week intervals between doses. Tumor development was monitored by palpation once a week for 20 weeks. If tumors were detected, their sizes were measured by calipers. The following endpoints were determined: incidence (number of animals with tumors), latency (time to first malignant tumor), multiplicity (number of all or malignant tumors per animal), and burden (total tumor volume per animal). When latency was assessed, mice that did not develop any tumors or only developed benign tumors were included in the analysis by assigning them a latency period of 21 weeks. Overall health of the mice was monitored daily. A mouse was euthanized prior to the end of the tumor monitoring period if it lost a significant amount of weight or if a tumor reached 10% of the animal’s body weight. Tumor histopathology was assessed by a certified pathologist who has expertise in studying mouse mammary tumors.

### Administration of hydralazine and valproic acid

One week following the final dose administration of DMBA, female mouse offspring from both control and HF *in utero* exposed groups were randomly selected to drink water containing valproic acid (Sigma, Milwaukee, WI) and hydralazine (Sigma, Milwaukee, WI), resulting in the daily intake of 1.2 g/kg VPA and 5 mg/kg hydralazine. VPA/hydralazine containing water packs were made fresh weekly. Administration of VPA/hydralazine containing water continued until the end of the study, which was 20 weeks post final DMBA administration.

### Tissue collection

At the end of tumor monitoring period, experimental animals were euthanized. We then collected mammary glands and tumors, and resected a portion of tumors for formalin-fixed paraffin embedding, and flash froze the remaining tissues in cryotubes in liquid nitrogen.

### RNA extraction and cDNA synthesis

Total RNA was extracted from the mammary glands and tumors using the Qiagen RNeasy Mini kit (Qiagen, MD) per the manufacturer’s protocol. Concentration and purity of the extracted RNA was determined by Nanodrop 1000 spectrophotometer (Thermo Scientific, DE). RNA (2 μg) per sample was used to generate cDNA via reverse transcription using the High-Capacity cDNA Reverse Transcription Kit (Applied Biosystems, CA) and run on a PTC-100 thermal cycler (Bio-Rad, CA).

### Quantitative real-time polymerase chain reaction

Product cDNA was brought to a working concentration of 5 ng/μL and mixed with EvaGreen 2X qPCR MasterMix-ROX (ABM, INC.) and gene specific forward and reverse primers. Primers used in qPCR analysis were designed using IDT tool primer design (Integrated DNA Technologies, IA, primer sequence found in Supplemental Table 1). Real-time qPCR was carried out using a 7900HT Real-Time PCR system (Applied Biosystems, CA). Expression of target genes was calculated by the Relative Standard Curve Method normalized to the housekeeping gene *Tbp* for mouse tissue.

### Protein isolation and immunoblotting

Protein levels were assessed by Western blot in mammary glands and tumors. Total protein was extracted from tissues using RIPA lysis buffer (0.1% SDS, 0.5% Sodium Doxycholate, 1% NP-40, 1 mM EDTA, 1 mM sodium orthvandate, 1 mM PMSF, 5 mM pyrophosphate, 10 mM glycocerophosphate, 50 mM Tris-HCl pH 7.4, 150 mM NaCl) supplemented with Mini Complete Protease Inhibitor (Roche, Germany). Protein concentration was measured using the BCA Protein Assay kit (Thermo Scientific) per manufacturer’s protocol. Protein extracts were separated on a 4–12% gradient denaturing poly-acrylamide gel (SDS-PAGE). Proteins were then transferred to a nitrocellulose membrane using the Invitrogen iBlot 7-min Blotting System. This was followed by blocking with Tris buffered saline + Tween 20 (TBST) plus 5% nonfat dry milk and incubation with specific primary antibodies (1:1000) overnight at 4 °C (PERK: 3192-Cell Signaling; HIF-1α: NB 100–134-Novus Biologicals; p62: 5114-Cell Signaling; NFκB p65: sc-8008- Santa Cruz Biotechnology, DNMT1: 5032-Cell Signaling; DNMT3a: 2160-Cell signaling; HDAC1: 10197–1-AP-Proteintech; ERα: 21244-1-AP-Proteintech; ERβ: 14007-1-AP-Proteintech). After several washes in TBST, membranes were incubated with secondary antibody at room temperature for 1 hour. Membranes were developed using HyGLO Chemiluminescent HRP antibody detection spray and developed on an Amersham imaging system or exposed to Kodak autoradiography films. Protein levels were determined by band intensity using Quantity One software (Bio-Rad) and the target proteins were normalized by β-actin (1:1000, sc1616-Santa Cruz Biotechnology) or Cyclophilin (1:1000, 2175-Cell Signaling)

### DNA methylation

The DNA methylation analysis was performed with the targeted Next Generation Bisulfite Sequencing (tNGBS) by EpigenDx, Inc. DNA was extracted from 10 mg of mammary tumors using M-digestion Buffer (1×; ZymoResearch, CA) and proteinase K (ZymoResearch, CA) (20 mg/ml). The lysate was incubated for 2 hours at 65 °C, frozen and sent to EpigenDx. tNGBS was designed to cover all CpGs sites within about 4000 bases in the regions of interest. Region from −1500 to +1000 including promoter #1 and exon 1, and gene body region from +10000 to +15000 including intron 1, intron 2 and promoter #2 were covered (Supplementary Fig. 6).

### Statistical analysis

Kaplan-Meier survival curves were used to assess differences in tumor incidence between groups, followed by log rank test. Tumor latency and multiplicity differences were assessed by 2-way ANOVA, and Tukey post-hoc test was used to assess differences among two groups. Difference in tumor burden was assessed by Two-Way repeated measure ANOVA followed by Holm-Sidak post-hoc test. Differential gene/protein expression and DNA methylation among groups was assessed by Two-Way ANOVA followed by Holm-Sidak post-hoc test. The differences were considered statistically significant when p values were equal to or less than 0.05.

### Ethics approval

All animal procedures were approved by the Georgetown University Animal Care and Use Committee (GUACUC).

## Supplementary information

Supplementary material.

## Data Availability

The data sets supporting the conclusions of this study are included within the article and its Supplementary Files.

## References

[1] Carpenter DO, Bushkin-Bedient S (2013). Exposure to Chemicals and Radiation During Childhood and Risk for Cancer Later in Life. J. Adolesc. Health.

[2] Li CI (2010). Relationship between radiation exposure and risk of second primary cancers among atomic bomb survivors. Cancer Res..

[3] O’Keefe EP (2013). siRNAs and shRNAs: tools for protein knockdown by gene silencing. Mater. Methods.

[4] Carmichael A, Sami AS, Dixon JM (2003). Breast cancer risk among the survivors of atomic bomb and patients exposed to therapeutic ionising radiation. Eur. J. Surg. Oncol..

[5] Nathanson KL, Wooster R, Weber BL (2001). Breast cancer genetics: what we know and what we need. Nat. Med..

[6] Palmer JR (2006). Prenatal diethylstilbestrol exposure and risk of breast cancer. Cancer Epidemiol. Biomarkers Prev..

[7] Hoover RN (2011). Adverse health outcomes in women exposed in utero to diethylstilbestrol. N. Engl. J. Med..

[8] Cohn BA (2015). DDT Exposure in Utero and Breast Cancer. J. Clin. Endocrinol. Metab..

[9] de Assis S, Galam K, Hilakivi-Clarke L (2006). High birth weight increases mammary tumorigenesis in rats. Int. J. Cancer.

[10] Michels,K. B. & Xue, F. Role of birthweight in the etiology of breast cancer. *Int J Cancer* (2006). 10.1002/ijc.22004 16823839

[11] Stavola BL (2000). Birthweight, childhood growth and risk of breast cancer in a British cohort. Br. J. Cancer.

[12] Hilakivi-Clarke L (1997). A maternal diet high in n-6 polyunsaturated fats alters mammary gland development, puberty onset, and breast cancer risk among female rat offspring. Proc. Natl Acad. Sci. USA.

[13] de Assis S (2012). High-fat or ethinyl-oestradiol intake during pregnancy increases mammary cancer risk in several generations of offspring. Nat. Commun..

[14] Nguyen N (2017). Maternal intake of high n-6 polyunsaturated fatty acid diet during pregnancy causes transgenerational increase in mammary cancer risk in mice. Breast Cancer Res..

[15] Walker BE (1990). Tumors in female offspring of control and diethylstilbestrol-exposed mice fed high-fat diets. J. Nat. Cancer Inst..

[16] Luijten M (2004). Effects of soy-derived isoflavones and a high-fat diet on spontaneous mammary tumor development in Tg.NK (MMTV/c-neu) mice. Nutr. Cancer.

[17] Leung YK (2017). Gestational high-fat diet and bisphenol A exposure heightens mammary cancer risk. Endocr. Relat. Cancer.

[18] Montales MT, Melnyk SB, Simmen FA, Simmen RC (2014). Maternal metabolic perturbations elicited by high-fat diet promote Wnt-1-induced mammary tumor risk in adult female offspring via long-term effects on mammary and systemic phenotypes. Carcinogenesis.

[19] Marks KJ (2017). Exposure to phytoestrogens in utero and age at menarche in a contemporary British cohort. Environ. Res..

[20] Tamimi RM (2016). Population Attributable Risk of Modifiable and Nonmodifiable Breast Cancer Risk Factors in Postmenopausal Breast Cancer. Am. J. Epidemiol..

[21] Walker CL, Ho SM (2012). Developmental reprogramming of cancer susceptibility. Nat. Rev. Cancer.

[22] Felsenfeld, G. A brief history of epigenetics. *Cold Spring Harb*. *Perspect*. *Biol*. **6** (2014). 10.1101/cshperspect.a018200 PMC3941222 24384572

[23] Dworkin AM, Huang TH, Toland AE (2009). Epigenetic alterations in the breast: Implications for breast cancer detection, prognosis and treatment. Semin. Cancer Biol..

[24] Basse C, Arock M (2015). The increasing roles of epigenetics in breast cancer: Implications for pathogenicity, biomarkers, prevention and treatment. Int. J. Cancer.

[25] Yang Y, Yin W, Wu F, Fan J (2017). Combination of azacitidine and trichostatin A decreased the tumorigenic potential of lung cancer cells. Onco. Targets. Ther..

[26] Capobianco E (2014). Separate and combined effects of DNMT and HDAC inhibitors in treating human multi-drug resistant osteosarcoma HosDXR150 cell line. PLoS One.

[27] Sato T (2017). Transcriptional Selectivity of Epigenetic Therapy in Cancer. Cancer Res..

[28] Gnyszka A, Jastrzebski Z, Flis S (2013). DNA methyltransferase inhibitors and their emerging role in epigenetic therapy of cancer. Anticancer. Res..

[29] Therasse P (2000). New guidelines to evaluate the response to treatment in solid tumors. European Organization for Research and Treatment of Cancer, National Cancer Institute of the United States, National Cancer Institute of Canada. J. Natl. Cancer Inst..

[30] Gottlicher M (2001). Valproic acid defines a novel class of HDAC inhibitors inducing differentiation of transformed cells. EMBO J..

[31] Phiel CJ (2001). Histone deacetylase is a direct target of valproic acid, a potent anticonvulsant, mood stabilizer, and teratogen. J. Biol. Chem..

[32] Singh N, Duenas-Gonzalez A, Lyko F, Medina-Franco JL (2009). Molecular modeling and molecular dynamics studies of hydralazine with human DNA methyltransferase 1. ChemMedChem..

[33] Mani E, Medina LA, Isaac-Olive K, Duenas-Gonzalez A (2014). Radiosensitization of cervical cancer cells with epigenetic drugs hydralazine and valproate. Eur. J. Gynaecol. Oncol..

[34] Cervera E (2012). Epigenetic therapy with hydralazine and magnesium valproate reverses imatinib resistance in patients with chronic myeloid leukemia. Clin. Lymphoma Myeloma. Leuk..

[35] Bauman J (2014). A Phase I Protocol of Hydralazine and Valproic Acid in Advanced, Previously Treated Solid Cancers. Transl. Oncol..

[36] Duenas-Gonzalez A (2014). Hydralazine-valproate: a repositioned drug combination for the epigenetic therapy of cancer. Expert. Opin. Drug. Metab. Toxicol..

[37] Arce C (2006). A proof-of-principle study of epigenetic therapy added to neoadjuvant doxorubicin cyclophosphamide for locally advanced breast cancer. PLoS. ONE..

[38] Hilakivi-Clarke Leena, Wärri Anni, Bouker Kerrie B., Zhang Xiyuan, Cook Katherine L., Jin Lu, Zwart Alan, Nguyen Nguyen, Hu Rong, Cruz M. Idalia, de Assis Sonia, Wang Xiao, Xuan Jason, Wang Yue, Wehrenberg Bryan, Clarke Robert (2016). Effects of In Utero Exposure to Ethinyl Estradiol on Tamoxifen Resistance and Breast Cancer Recurrence in a Preclinical Model. Journal of the National Cancer Institute.

[39] Alsdorf R, Wyszynski DF (2005). Teratogenicity of sodium valproate. Expert. Opin. Drug. Saf..

[40] Sestak I (2018). Comparison of the Performance of 6 Prognostic Signatures for Estrogen Receptor-Positive Breast Cancer: A Secondary Analysis of a Randomized Clinical Trial. JAMA Oncol..

[41] Biton V (2001). Weight change associated with valproate and lamotrigine monotherapy in patients with epilepsy. Neurology.

[42] Chengappa KN (2002). Changes in body weight and body mass index among psychiatric patients receiving lithium, valproate, or topiramate: an open-label, nonrandomized chart review. Clin. Ther..

[43] Wu HC (2018). Breast cancer family history and allele-specific DNA methylation in the legacy girls study. Epigenetics..

[44] Yang X (2001). Synergistic activation of functional estrogen receptor (ER)-alpha by DNA methyltransferase and histone deacetylase inhibition in human ER-alpha-negative breast cancer cells. Cancer Res..

[45] Jones PA (2012). Functions of DNA methylation: islands, start sites, gene bodies and beyond. Nat. Rev. Genet..

[46] Arechederra M (2018). Hypermethylation of gene body CpG islands predicts high dosage of functional oncogenes in liver cancer. Nat. Commun..

[47] Walter P, Ron D (2011). The unfolded protein response: from stress pathway to homeostatic regulation. Science.

[48] Xu LZ (2017). p62/SQSTM1 enhances breast cancer stem-like properties by stabilizing MYC mRNA. Oncogene.

[49] Puvirajesinghe TM (2016). Identification of p62/SQSTM1 as a component of non-canonical Wnt VANGL2-JNK signalling in breast cancer. Nat. Commun..

[50] Luo RZ (2013). Accumulation of p62 is associated with poor prognosis in patients with triple-negative breast cancer. Onco. Targets. Ther..

[51] Birgisdottir V (2006). Epigenetic silencing and deletion of the BRCA1 gene in sporadic breast cancer. Breast Cancer Res..

[52] Saunderson EA (2017). Hit-and-run epigenetic editing prevents senescence entry in primary breast cells from healthy donors. Nat. Commun..

[53] Lu YM, Cheng F, Teng LS (2016). The association between phosphatase and tensin homolog hypermethylation and patients with breast cancer, a meta-analysis and literature review. Sci. Rep..

[54] Segura-Pacheco B (2003). Reactivation of tumor suppressor genes by the cardiovascular drugs hydralazine and procainamide and their potential use in cancer therapy. Clin. Cancer Res..

[55] Zambrano P (2005). A phase I study of hydralazine to demethylate and reactivate the expression of tumor suppressor genes. BMC. Cancer.

[56] Yang X (2014). Gene body methylation can alter gene expression and is a therapeutic target in cancer. Cancer Cell.

[57] Kimbro KS, Simons JW (2006). Hypoxia-inducible factor-1 in human breast and prostate cancer. Endocr. Relat. Cancer.

[58] Xia Y, Shen S, Verma IM (2014). NF-kappaB, an active player in human cancers. Cancer Immunol. Res..

[59] Rozpedek W (2016). The Role of the PERK/eIF2alpha/ATF4/CHOP Signaling Pathway in Tumor Progression During Endoplasmic Reticulum Stress. Curr. Mol. Med..

[60] Moscat J, Karin M, Diaz-Meco MT (2016). p62 in Cancer: Signaling Adaptor Beyond Autophagy. Cell.

[61] Knowles HJ, Tian YM, Mole DR, Harris AL (2004). Novel mechanism of action for hydralazine: induction of hypoxia-inducible factor-1alpha, vascular endothelial growth factor, and angiogenesis by inhibition of prolyl hydroxylases. Circ. Res..

[62] Cubillos-Ruiz JR, Bettigole SE, Glimcher LH (2017). Tumorigenic and Immunosuppressive Effects of Endoplasmic Reticulum Stress in. Cancer. Cell.

[63] Hallas J (2009). Cancer risk in long-term users of valproate: a population-based case-control study. Cancer Epidemiol. Biomarkers Prev..

[64] Brodie SA, Brandes JC (2014). Could valproic acid be an effective anticancer agent? The evidence so far. Expert. Rev. Anticancer. Ther..

[65] Zhang Y, Kutateladze TG (2018). Diet and the epigenome. Nat. Commun..

[66] Lewis KA, Tollefsbol TO (2017). The influence of an epigenetics diet on the cancer epigenome. Epigenomics..

