# Supplementary material for: Reversal of increased mammary tumorigenesis by valproic acid and hydralazine in offspring of dams fed high fat diet during pregnancy
Source: Sci Rep. 2019 Dec 30;9:20271. doi: 10.1038/s41598-019-56854-5 (PMC6937280; doi:10.1038/s41598-019-56854-5)
Supplement: Supplementary file 1 — Supplementary material. [file 41598_2019_56854_MOESM1_ESM.pdf]

Reversal of increased mammary tumorigenesis by valproic acid and hydralazine in offspring of dams fed high fat diet during pregnancy

de Oliveira Andrade F, Nguyen NM, Warri A, and Hilakivi-Clarke L

Supplemental Table 1. Primers used

Gene ID	Sequence
Brca1 _forward	5’-AGGGAAGCACAAGGTTTAGTC-3’
Brca1 _reverse	5’-CCTCATTCAAACGCTCACAAG-3’
Cdkn2a _forward	5’-GAACTCTTTCGGTCGTACCC-3’
Cdkn2a _reverse	5’-CAGTTCGAATCTGCACCGTAG-3’
Igfbp6 _ forward	5’- GCTCTATGTGCCAAACTGTG -3’
*Igfbp6*_ reverse	5’-TGAGTGCTTCCTTGACCATC -3’
Oas3a _forward	5’-GCCTGCTTTTGATGCTGTG-3’
Oas3a _reverse	5’-GGAGGGCAAGTGTTTATGAAG-3’
p21 _forward	5’-CTGTCTTGCACTCTGGTGTCTGA-3’
p21 _reverse	5’-CCAATCTGCGCTTGGAGTGA-3’
Slfn1 _forward	5’-CATAGAGGAATGGATCAAGCTCC-3’
Slfn1 _reverse	5’-AAACCCTTCCAACATCCCC-3’
Tbp _forward	5’-AGGATGCTCTAGGGAAGATCTGAG-3’
Tbp _reverse	5’-GAGCATAAGGTGGAAGGCTGTT-3’
Zbp1 _ forward	5’- GGACAGACGTGGAAGATCTAC -3’
*Zbp1*_ reverse	5’- ATGGAGATGTGGCTGTTGG -3’

Supplementary figures

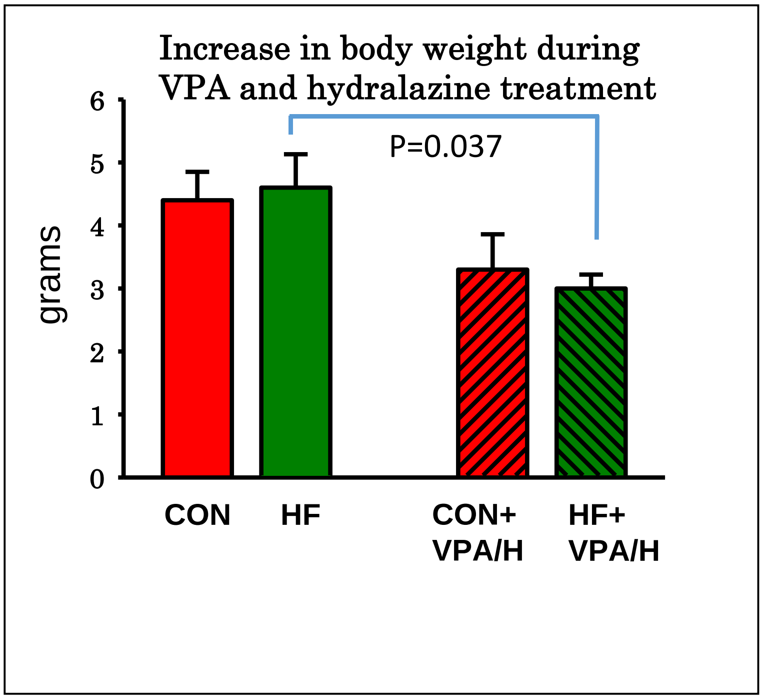

Supplementary Figure 1. Body weight gain during valproic acid (VPA) and hydralazine treatment. No changes in body weight gains were seen between control (CON) and high fat (HF) offspring. However, mice receiving VPA/hydralazine gained less weight than non-treated mice (p=0.018 for treatment). P-value obtained in Holm-Sidak post-hoc test is shown in figure. Weight gains were assessed from starting VPA/hydralazine treatment to the end of tumor monitoring period. Means and SEM of 18-30 mice per group are shown.

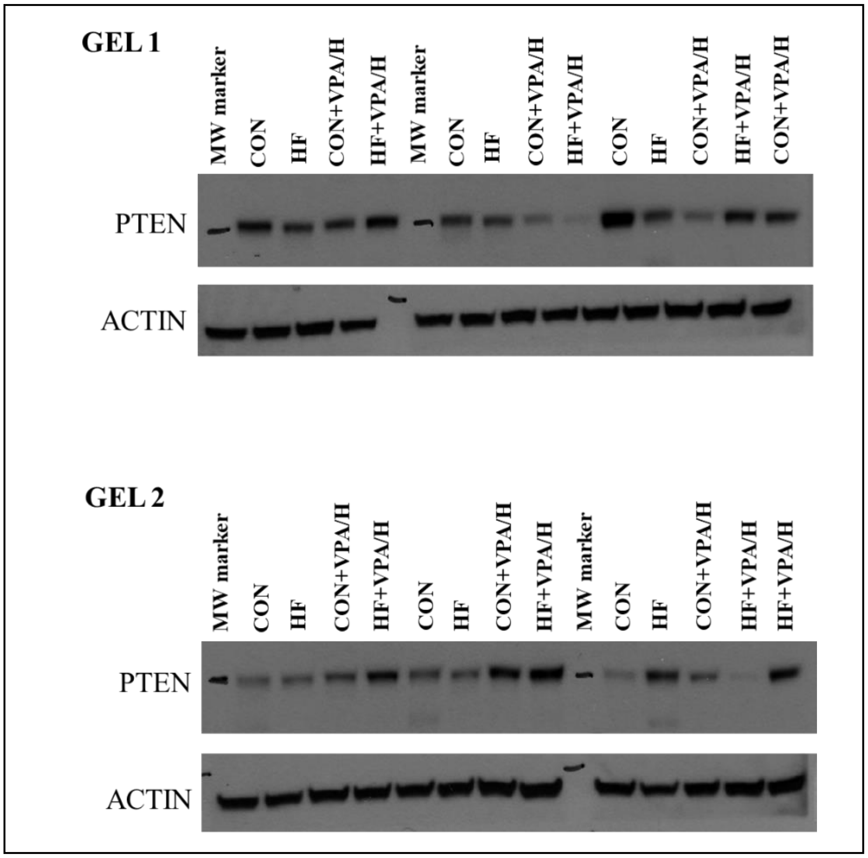

Supplementary Figure 2. Full-length blot of PTEN and ACTIN. After transfer, membrane was cropped to incubate with anti-PTEN and anti-ACTIN primary antibodies in the same western blot. Incubation and development was done together for the correspondent protein from gel 1 and gel 2.

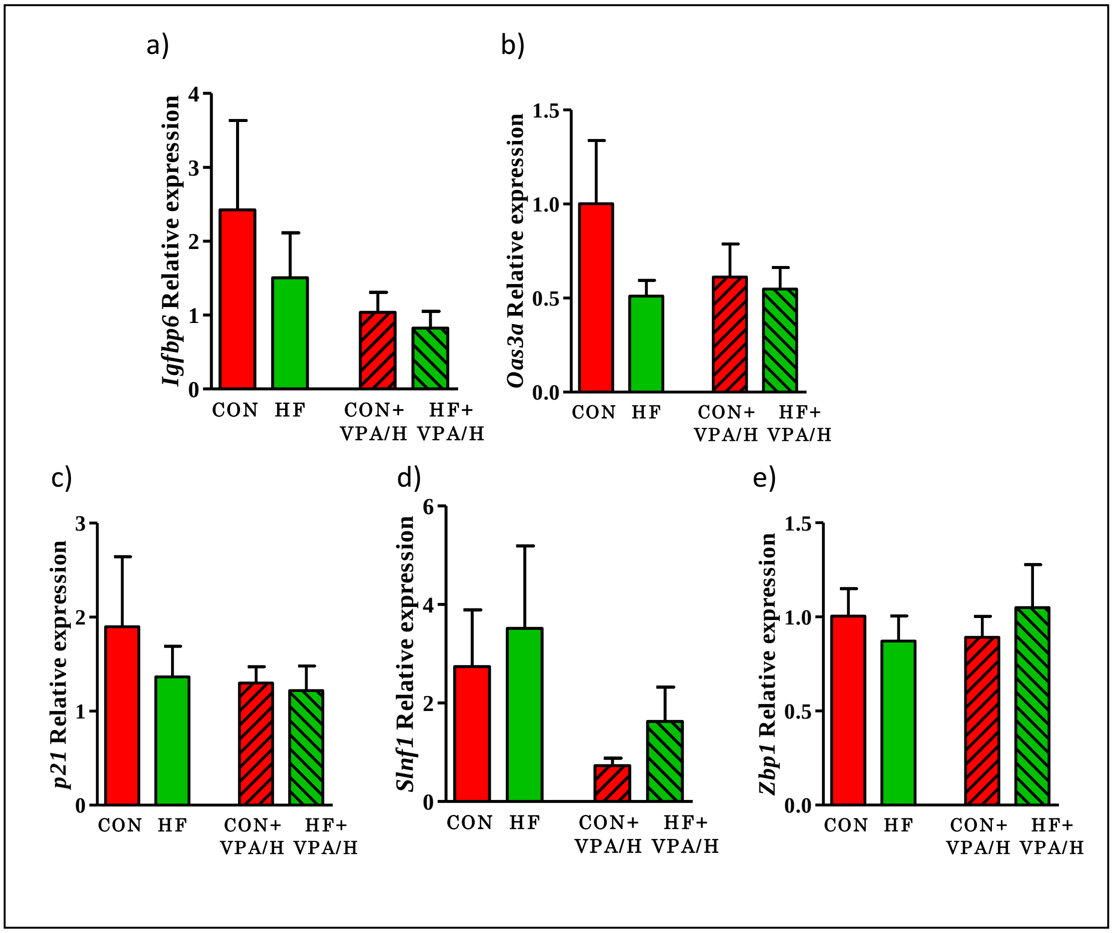

Supplementary Figure 3. Effect of valproic acid (VPA) and hydralazine on tumor suppressor gene expression in mammary tumors. *In utero* HF exposure did not change the expression of a) *Igfbp6*, b) *Oas3a*, c) *p21*, *d*) *Slfn1* and e) *Zbp1*. Treatment with VPA/hydralazine did not modify the expression of these genes either. Means and SEM of 5-7 mammary tumors per group are shown.

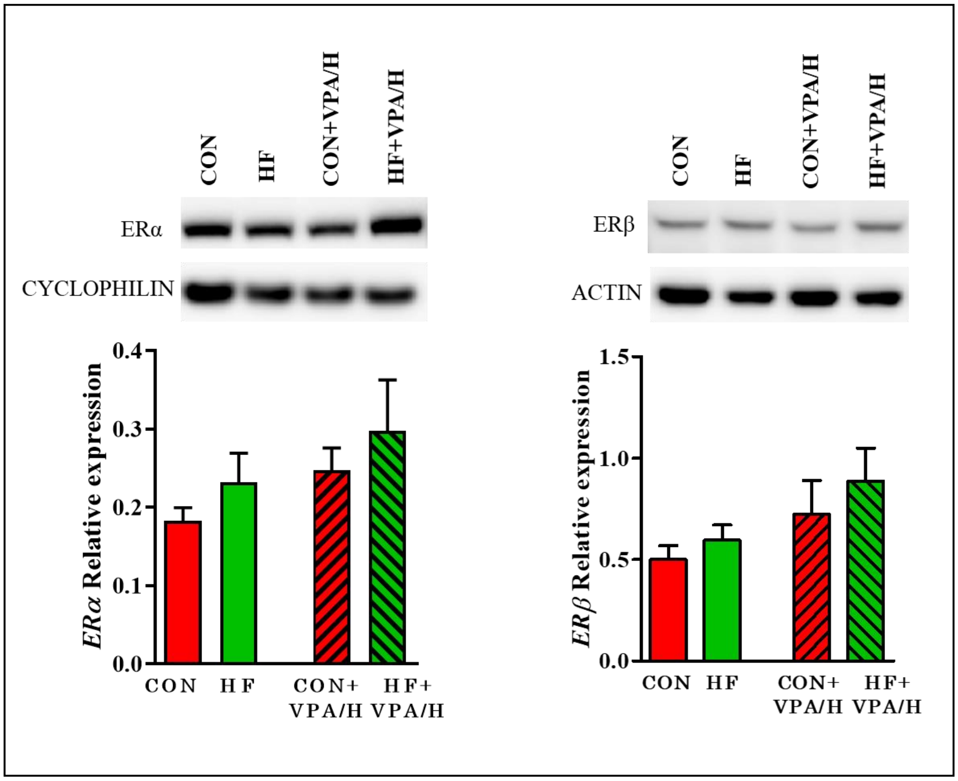

Supplementary Figure 4. Effect of valproic acid (VPA) and hydralazine on ERα and ERβ protein levels in mammary tumors. Treatment with VPA/hydralazine did not significantly modify the expression of ERα or ERβ. Means and SEM of 5-7 mammary tumors per group are shown.

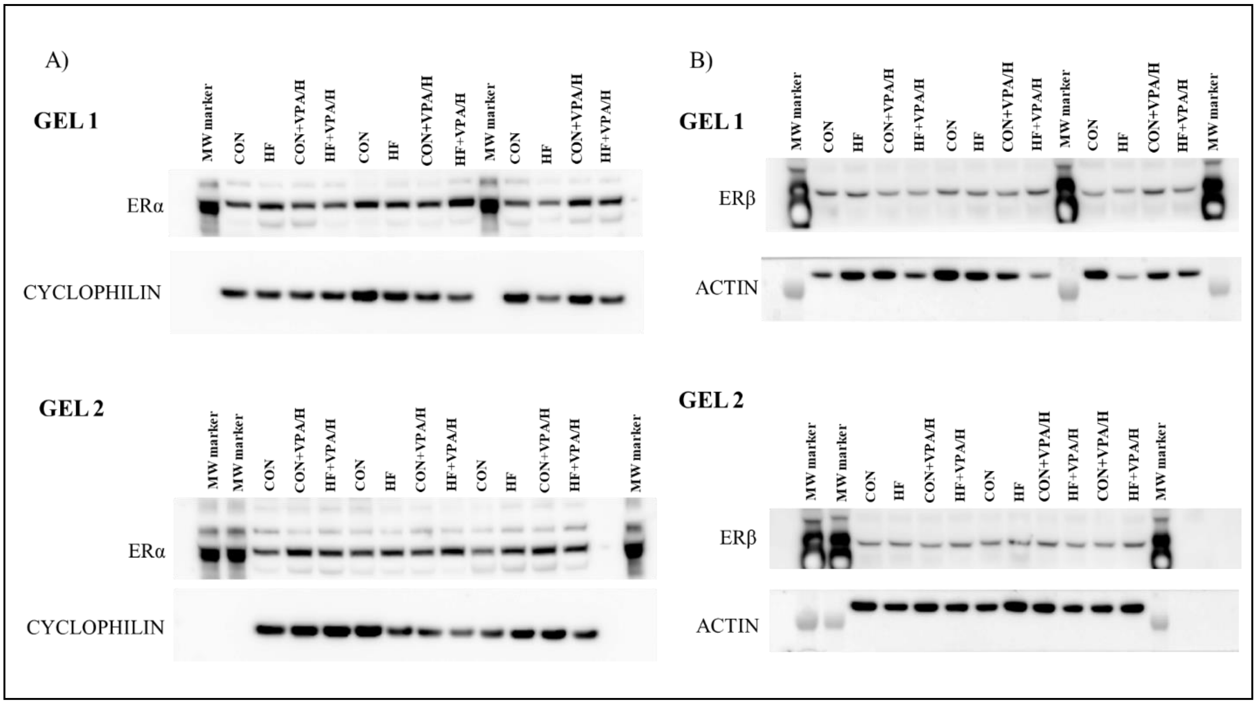

Supplementary Figure 5. Full-length blot of ERα ERβ, CYCLOPHILIN and ACTIN. After transfer, membrane was cropped to incubate with A) anti- ERα and anti-CYCLOPHILIN primary antibodies in the same western blot or B) anti-ERβ primary antibody. After development of ERβ, the membrane was incubated with Restore™ Western Blot Stripping Buffer (ThermoFisher Scientific) for 15 minutes, blocked with Tris buffered saline +Tween 20 (TBST) plus 5% nonfat dry milk for 1hr and incubated overnight with anti-ACTIN. Stripping was confirmed developing the membrane on an Amersham imaging system. Incubation and development was done together for the correspondent protein from gel 1 and gel 2.

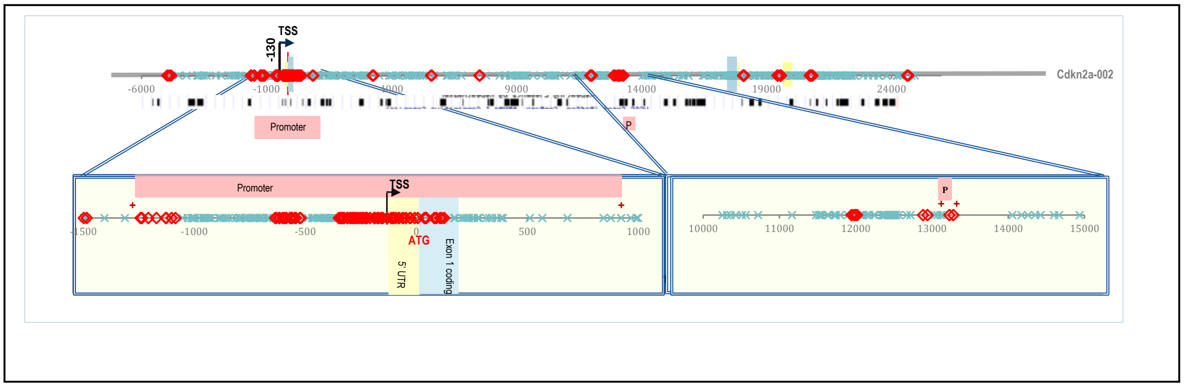

Supplementary Figure 6. Study design to assess methylation of CpG islands in mouse Cdkn2a/p16 gene. DNA methylation analysis was performed with the targeted Next Generation Bisulfite Sequencing (tNGBS). *Cdkn2a* region from -1500 to + 1000 including promoter #1 and exon 1, and gene body region from +10000 to +15000 including intron 1, intron 2 and promoter #2 were covered. Red diamonds are the CpG sites sequenced.

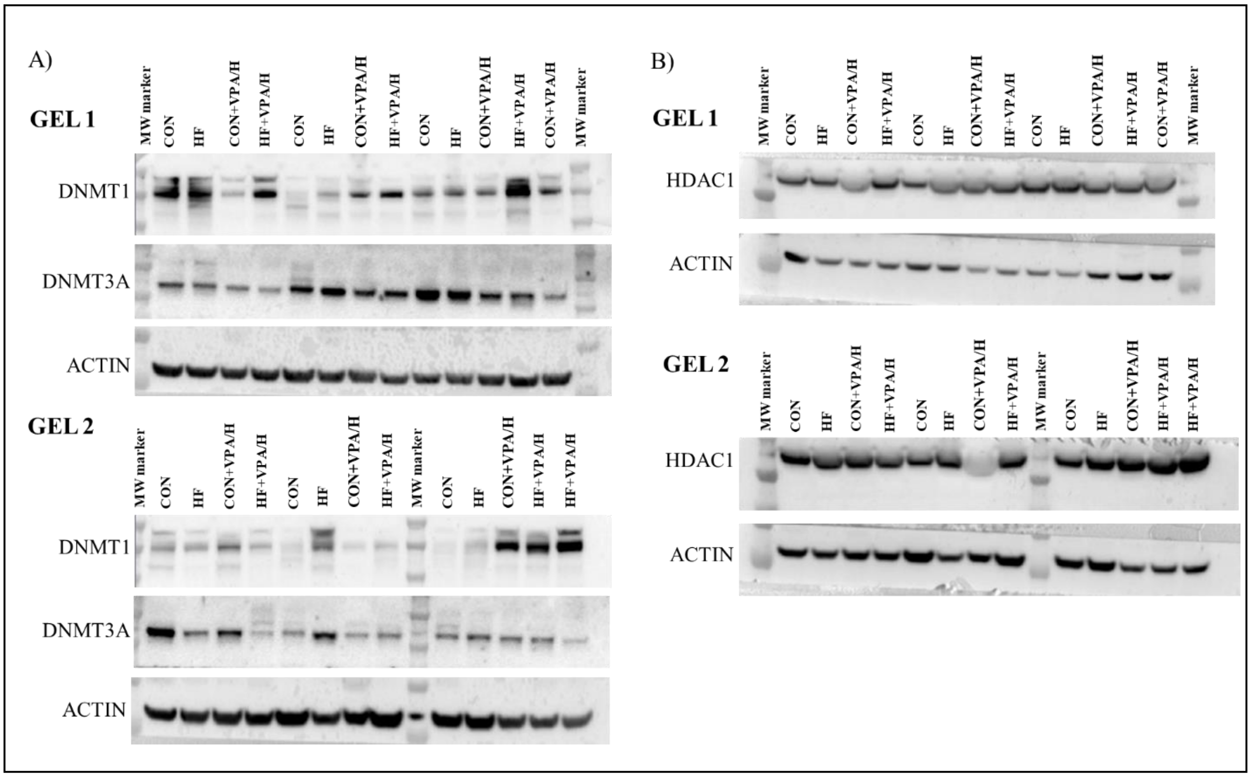

Supplementary Figure 7: Full-length blot of DNMT1, DNMT3A, HDAC1 and ACTIN. After transfer, membrane was cropped to incubate with A) anti- DNMT3A and anti-ACTIN and B) HDAC1 and ACTIN primary antibodies in the same western blot. After development of DNMT3A, the membrane was incubated with Restore™ Western Blot Stripping Buffer (ThermoFisher Scientific) for 15 minutes, blocked with Tris buffered saline +Tween 20 (TBST) plus 5% nonfat dry milk for 1hr and incubated overnight with anti-DNMT1. Stripping was confirmed developing the membrane on an Amersham imaging system. Incubation and development were done together for the correspondent protein from gel 1 and gel 2.

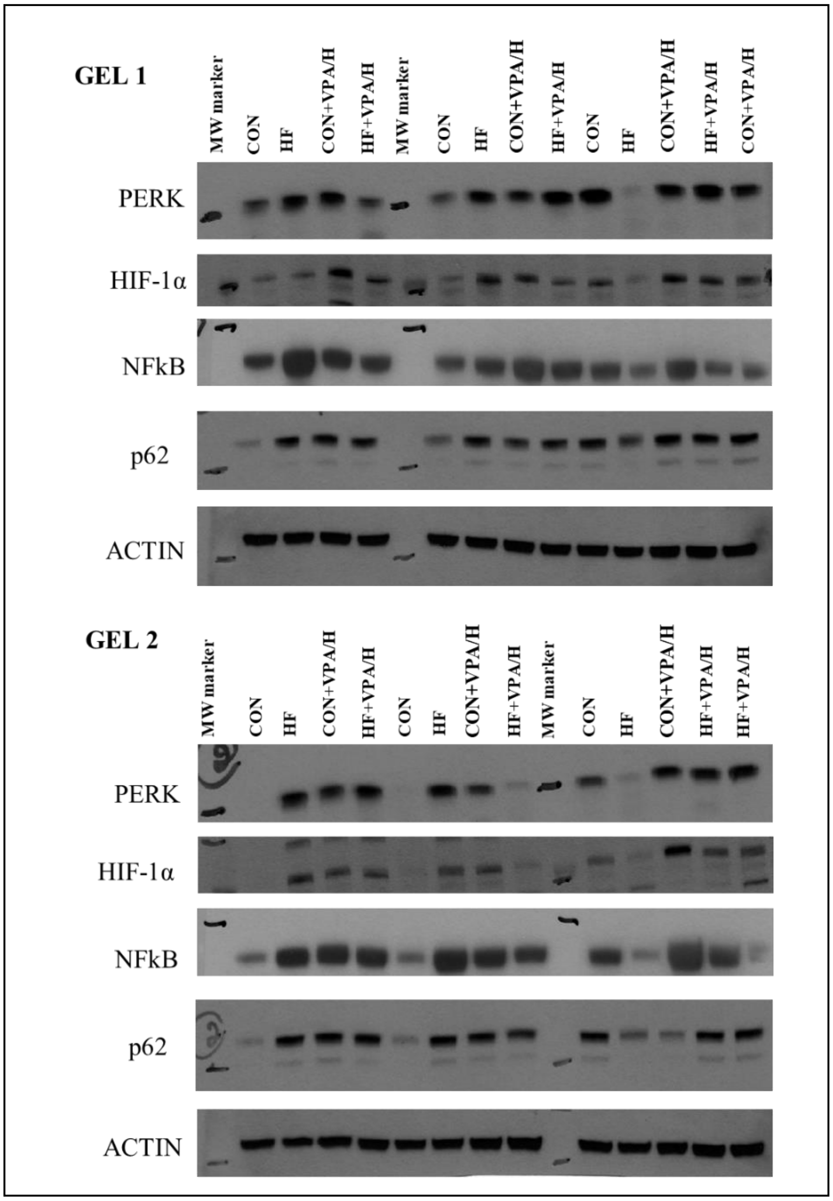

Supplementary Figure 8: Full-length blot of PERK, HIF-1α,NFkB, p62 and ACTIN. After transfer, membrane was cropped to incubate with anti-PERK, anti-HIF-1^{α} and anti-p62 primary antibodies in the same western blot. After development of p62, the membrane was incubated with Restore™ Western Blot Stripping Buffer (ThermoFisher Scientific) for 15 minutes, blocked with Tris buffered saline +Tween 20 (TBST) plus 5% nonfat dry milk for 1hr and incubated overnight with anti-NFkB primary antibody. The procedure was repeated after development of NFkB to incubate membrane with anti-ACTIN. Stripping was confirmed developing the membrane on an Amersham imaging system. Incubation and development were done together for the correspondent protein from gel 1 and gel 2.
